# Supplementary material for: Pulmonary Tuberculosis and Risk of Lung Cancer: A Systematic Review and Meta-Analysis
Source: J Clin Med. 2022 Jan 30;11(3):765. doi: 10.3390/jcm11030765 (PMC8836400; doi:10.3390/jcm11030765)
Supplement: Supplementary file 1 [file jcm-11-00765-s001.zip › jcm-1565354-supplementary.pdf]

Supplementary material

Table S1. Full search strategy. Search was performed on August 30, 2020.

**PubMed**
(tb[tiab] OR tuberculo*[tiab] OR mycobacterium[tiab] OR "Tuberculosis"[Mesh])<br>AND<br>("Lung cancer*"[tiab] OR "Lung tumor*"[tiab] OR "Lung tumour*"[tiab] OR "lung neoplasm*"[tiab] OR "lung carcinoma"[tiab] OR NSCLC[tiab] OR SCLC[tiab] OR "lung adenocarcinoma"[tiab] OR "Lung Neoplasms"[Mesh])<br>NOT<br>("review"[pt] OR "Case Reports"[pt] OR "Clinical Trial"[pt] OR "Meta-Analysis"[pt] OR "Systematic Review"[pt] OR "letter"[pt] OR "editorial"[pt] OR "news"[pt])
Result: 4,339 hits
**EMBASE**
(tb:ti,ab,kw OR "tuberculosis"/exp OR tuberculo*:ti,ab,kw)<br>AND<br>("lung cancer*":ti,ab,kw OR "lung tumor"/exp OR "lung tumor*":ti,ab,kw OR "lung tumour*":ti,ab,kw OR "lung neoplasm*":ti,ab,kw OR "lung carcinoma":ti,ab,kw OR "NSCLC":ti,ab,kw OR "non-small cell lung cancer"/exp OR "SCLC":ti,ab,kw OR "small cell lung cancer"/exp OR "lung adenocarcinoma":ti,ab,kw OR "lung adenocarcinoma"/exp)<br>NOT<br>("animal"/exp NOT "human"/exp)<br>NOT<br>(("controlled clinical trial"/de OR "meta-analysis"/de OR "systematic review"/de OR "clinical trial"/de OR "case report"/de OR "diagnostic test accuracy study"/de) NOT ("cohort analysis"/de OR "cross sectional study"/de OR "observational study"/de OR "prospective study"/de OR "retrospective study"/de))<br>NOT ("conference abstract":it OR "conference paper":it OR "conference review":it OR editorial:it OR note:it OR letter:it OR "short survey":it OR "review":it)
Result: 5,695 hits
**Cochrane Database**
(tb OR tuberculo* OR mycobacterium)<br>AND<br>("Lung cancer*" OR "Lung tumor*" OR "Lung tumour*" OR "lung neoplasm*" OR "lung carcinoma" OR NSCLC OR SCLC OR "lung adenocarcinoma")
Result: 135 hits

Table S2. Meta-regression analysis between pulmonary tuberculosis and lung cancer

Continuous variable	All studies (n=33)			High-quality studies (n=8)		
	Regression coefficient	*p*-value	No. of studies	Regression coefficient	*p*-value	No. of studies
Mean age, years	0.949	< 0.001	27	0.945	< 0.001	7
Female, %	0.999	0.975	31	1.001	0.949	8
SCLC, %	0.991	0.302	15	_	_	0
LCLC, %	0.965	0.263	13	_	_	0
Adenocarcinoma, %	0.996	0.367	17	_	_	0
Squamous cell cancer, %	0.995	0.532	16	_	_	0
Diabetes, %	_	_	0	_	_	0
Hypertension, %	_	_	0	_	_	0
COPD, %	0.983	0.196	19	0.970	0.260	7
Non-smoker, %	1.006	0.115	17	1.008	0.156	4
Ever smoker, %	0.994	0.115	17	0.993	0.156	4

Abbreviations: COPD, chronic obstructive pulmonary diseases; LCLC, large cell lung cancer; No, number; SCLC, small cell lung cancer.
